# Iron deficiency in heart failure: Epidemiology, diagnostic criteria and treatment modalities

**DOI:** 10.1002/ehf2.15157

**Published:** 2024-10-30

**Authors:** Stephan von Haehling

**Affiliations:** ^1^ Department of Cardiology and Pneumology University Göttingen Medical Center (UMG) Göttingen Germany; ^2^ DZHK (German Centre for Cardiovascular Research), partner site Lower Saxony Göttingen Germany

Iron deficiency has received increasing attention in recent years in heart failure because it has been associated with reduced exercise capacity, reduced quality of life and increased morbidity and mortality. The purpose of this *Virtual Issue* of ESC Heart Failure is to highlight a number of studies that have been published in the *Journal* to shed further light on this important topic.

## Epidemiology

Sharma *et al*. conducted a case cohort study with 1006 participants from the Cardiovascular Health Study, all aged 64 years and older, who did not have heart failure at baseline. The study aimed to evaluate the associations between iron status and the incidence of heart failure. Participants were categorized into quartiles of transferrin saturation (TSAT) and ferritin levels and classified as iron replete (27.3%), having functional iron deficiency (7.7%), iron deficiency (11.8%), mixed iron deficiency (5.6%), high iron status (9.3%) or non‐classified (31.1%). After adjusting for demographics, heart failure risk factors and estimated glomerular filtration rate, the study found that older adults with iron deficiency had a higher risk of developing heart failure (hazard ratio 1.47, 95% confidence interval 1.02–2.11) compared to those without iron deficiency.^1^ Cabrera *et al*. investigated the prevalence of iron deficiency in newly diagnosed heart failure patients and tracked the progression of iron deficiency parameters after the initiation of heart failure therapy. This prospective cohort study was conducted across five hospitals in Sweden. Among 482 patients with complete iron data at baseline, 163 (34%) had iron deficiency, defined according to the European Society of Cardiology (ESC) criteria (ferritin <100 μg/L or ferritin 100–299 μg/L with TSAT <20%). A similar prevalence was observed after 12 months, with 119 out of 368 patients (32%) having iron deficiency. During the first year following a heart failure diagnosis, 19% had persistent iron deficiency, 13% developed iron deficiency, 11% resolved iron deficiency, and 57% never had iron deficiency. Overall, 24% of patients did not change their classification. Baseline anaemia was the strongest independent predictor of prevalent iron deficiency 1 year after heart failure diagnosis. The authors concluded that about one‐third of patients with newly diagnosed heart failure had iron deficiency both at diagnosis and after 1 year of follow‐up.^2^ The CARENFER study assessed the prevalence of iron deficiency in a French cohort using the ESC's standard criteria for diagnosing iron deficiency. Sixty per cent of the patients had decompensated heart failure. The overall prevalence of iron deficiency was 49.6%, with higher rates observed during cardiac decompensation compared to patients with chronic heart failure (58.1% vs. 39.0%). Interestingly, the study found that patients with heart failure with preserved ejection fraction were more likely to have iron deficiency than those with mildly reduced or reduced left ventricular ejection fraction.^3^ In alignment with these findings, van Dalen *et al*. explored the prevalence and natural progression of iron deficiency in patients with acute heart failure. Using data from a prospective multicentre observational study that included 741 patients admitted with acute heart failure and applying the standard criteria for iron deficiency, they discovered that iron deficiency was prevalent in 71.8% of patients at baseline. Before discharge, the prevalence decreased to 56.4%, and 10 ± 6 weeks after discharge, it further decreased to 50.3%. Absolute iron deficiency persisted in 66% of patients from baseline to 10 ± 6 weeks of follow‐up, while functional iron deficiency resolved in 56% of patients. These findings reinforce the view that iron deficiency is highly prevalent in patients with acute heart failure and that it remains a significant issue even after re‐compensation.^4^

## Diagnostic criteria

A significant debate surrounds the correct diagnosis of iron deficiency in patients with heart failure. Graham *et al*. studied 4422 patients attending a clinic that served a large local population in the United Kingdom. They found that the lowest quartile of serum transferrin concentration (not TSAT) was associated with older age, lower serum iron concentration and haemoglobin, as well as higher levels of high‐sensitivity C‐reactive protein, ferritin and N‐terminal B‐type natriuretic peptide. Patients in the highest quartile of transferrin concentration were found to have TSAT values below 20% even when the serum iron concentration was higher than 13 μmol/L in 185 patients. The authors concluded that low serum transferrin concentration is frequently associated with low serum iron concentration, even when TSAT is >20% or serum ferritin is >100 μg/L. They also noted that these patients have a high prevalence of anaemia and a poor prognosis and might be iron‐deficient, even though they are currently excluded from clinical trials on iron depletion.^5^ In line with these findings, Tada *et al*. studied 763 patients with chronic heart failure from a Japanese multicenter registry. Using iron deficiency criteria with either TSAT <20% and serum iron ≤13 μmol/L or the guideline‐recommended iron deficiency criteria, the authors found that the prevalence of iron deficiency varied considerably. The prevalence was 28% using the newly proposed criteria and 58% using the guideline‐recommended criteria. During a follow‐up period of 436 days, 56 patients experienced all‐cause mortality events. Only the newly proposed iron deficiency criteria independently predicted all‐cause mortality on multivariable Cox regression. No such association was found using the guideline‐recommended criteria.^6^ The prevalence and determinants of iron deficiency in patients with cardiac amyloidosis were also studied in 816 patients enrolled at a French Referral Center for Cardiac Amyloidosis. Of these, 47% had wild‐type ATTR amyloidosis, and 33% had AL amyloidosis. Iron deficiency was present in 49% of all patients with cardiac amyloidosis. The most significant independent determinants of iron deficiency were ATTR status, diabetes, aspirin treatment, haemoglobin level and altered global longitudinal strain. No difference was detected in all‐cause mortality when iron deficiency status was considered.^7^

## Imaging and iron deficiency

In a post hoc sub‐analysis of the double‐blind, placebo‐controlled, randomized Myocardial‐IRON Trial, which included 53 ambulatory patients with heart failure and iron deficiency treated with either ferric carboxymaltose or placebo, significant improvements in cardiac magnetic resonance‐featured tracking strain were observed in those who received ferric carboxymaltose.^8^ Similarly, Gertler *et al*. analysed 24 patients with heart failure with reduced ejection fraction using T2* magnetic resonance imaging to assess iron content in the left ventricle, small and large intestines, spleen, liver, skeletal muscle and brain. In a non‐randomized, uncontrolled study, 12 patients with iron deficiency were treated with ferric carboxymaltose. The study found that, as indicated by higher T2* values, iron content was lower in the spleen and liver, and there was a trend towards lower cardiac receptor iron content in these patients. In those treated with ferric carboxymaltose, left ventricular iron content increased by 25.4%, while spleen and liver iron content increased by 46.4% and 18.2%, respectively. Iron content in skeletal muscle, brain, intestine and bone marrow remained unchanged.^9^ These findings are consistent with the known association between iron deficiency and reduced exercise tolerance and quality of life. Ohori *et al*. enrolled consecutive patients with heart failure and conducted a short physical performance battery to evaluate physical function. Iron deficiency was defined using standard criteria. Among the 562 patients with heart failure, 329 (58%) had iron deficiency, and 191 (34%) had low physical function. The authors found, using multivariable logistic regression, that TSAT as a continuous variable, but not iron deficiency itself, was a predictor of low physical function. Interestingly, the association between low TSAT and low physical function was not observed in heart failure patients who also had diabetes mellitus. The authors concluded that iron supplementation therapy might have limited impact in patients with diabetes mellitus.^10^ It is well known that iron deficiency is frequently associated with anaemia. Patients with anaemia tend to have worse outcomes, including increased hospitalization rates, decreased exercise tolerance and higher mortality rates. Selenoprotein P is a key transporter and functional biomarker of selenium, and Jujić *et al*. hypothesized that lower concentrations of selenoprotein P would be associated with the prevalence of anaemia. In a study of 320 patients hospitalized with heart failure, they found that selenoprotein P concentrations in the lowest quartile were associated with anaemia, haemoglobin levels and iron status. Anaemia was present in 42.9% of all patients, and selenoprotein P concentrations were positively associated with haemoglobin levels and negatively with transferrin receptor 1 concentrations.^11^ Matsue *et al*. conducted a pilot multicentre, open‐label, randomized controlled trial in 50 patients with heart failure complicated by chronic kidney disease and anaemia. Patients in this trial were randomized 1:1 to either daprodustat or a control group across seven sites in Japan. Daprodustat, a hypoxia‐inducible factor‐prolyl hydroxylase inhibitor, is intended for use in patients with heart failure and renal anaemia. This study aims to recruit patients for whom no safe and effective treatment is currently available, as the anaemia in this case is caused not by iron deficiency but by chronic kidney disease.^12^

## Treatment modalities

Docherty *et al*. conducted a post hoc analysis of the IRONMAN trial, which randomized patients with heart failure and iron deficiency, defined as either transferrin saturation (TSAT) <20% or ferritin <100 μg/L. This sub‐analysis revealed that among the 1137 patients randomized to receive ferric derisomaltose or usual care, only 29 (2.6%) were taking an SGLT2 inhibitor at baseline. Notably, the authors observed a trend towards a greater increase in haemoglobin levels among iron‐deficient patients receiving ferric derisomaltose who were also on SGLT2 inhibitors at baseline. Specifically, the mean haemoglobin increase from baseline was 1.3 (±1.2) grams/decilitre in those treated with ferric derisomaltose, compared to 0.1 (±0.7) g/dL in the usual care group. In patients not taking SGLT2 inhibitors, the corresponding increase was 0.6 (±0.9) g/dL.^13^ A prospective single‐centre registry study is currently planned, involving patients with heart failure with reduced ejection fraction (HFrEF) who have implanted cardiac electronic devices. Participants, who will receive intravenous ferric carboxymaltose for iron deficiency, will be followed at baseline, 3, 6 and 12 months. The primary endpoint of this study is the composite of iron‐related changes in blood markers, including haemoglobin <12 g/dL, ferritin >50 ng/L and TSAT >20%. The trial aims to assess the effect of ferric carboxymaltose on iron deficiency, with a particular focus on the arrhythmic burden post‐treatment.^14^ Ahmed *et al*. conducted a meta‐analysis of randomized clinical trials on intravenous (IV) iron therapy for heart failure and iron deficiency, incorporating data from 14 trials with a total of 6651 patients. The analysis showed that IV iron therapy significantly reduced the composite endpoint of first heart failure hospitalization or cardiovascular death compared to the control group. Additionally, IV iron therapy was associated with trends towards lower cardiovascular mortality, all‐cause mortality at 1 year and first hospitalization for heart failure, along with improved left ventricular ejection fraction (LVEF).^15^ Similarly, Sindone *et al*. performed a meta‐analysis of 12 randomized controlled trials, including 2381 patients with heart failure with reduced ejection fraction (HFrEF) and iron deficiency or iron deficiency anaemia. Their analysis demonstrated that IV iron carbohydrate therapy significantly reduced hospitalization for worsening heart failure and the composite of first hospitalization for worsening heart failure or death, although it did not significantly impact all‐cause mortality. Importantly, IV iron carbohydrate therapy also improved functional and exercise capacity compared to the control group, with no significant difference in adverse events between the treatment groups.^16^ In a retrospective multicentre study, López‐Vilella *et al*. investigated 565 consecutive outpatients diagnosed with heart failure. Over 5 years, these patients were treated with intravenous ferric carboxymaltose for iron deficiency, identified using standard criteria. Following treatment, ferritin, TSAT and haemoglobin levels increased by up to fivefold, 1.6‐fold and 1.1‐fold, respectively. The increase in ferritin and TSAT was more pronounced in patients with heart failure with preserved ejection fraction (HFpEF). Additionally, the percentage of patients with normalization of right ventricular function increased by 6.9 percentage points in those with HFpEF, compared to 6.4 percentage points in those with HFrEF (*P* < 0.0001).^17^ Further supporting these findings, Salah *et al*. conducted a systematic review and meta‐analysis of 10 randomized controlled trials involving 3438 patients. Their analysis found that IV iron significantly reduced the composite of cardiovascular mortality and first hospitalization for heart failure, as well as total hospitalizations for heart failure. However, no significant difference was observed in all‐cause mortality or cardiovascular mortality.^18^

One concern with ferric carboxymaltose treatment is the risk of hypophosphatemia. Rosano *et al*. conducted a pooled analysis of 41 clinical trials, including data from 7931 patients treated with ferric carboxymaltose across various disease states. Of these patients, 14% had heart failure, 36% had women's health conditions, 27% had non‐dialysis‐dependent chronic kidney disease (CKD), 1% had haemodialysis‐dependent CKD, 10% had gastrointestinal conditions, 3% had neurological conditions, and 10% had other conditions. The incidence of severe hypophosphatemia (serum phosphate <2.0 mg/dL) varied across therapeutic areas, with the lowest incidence observed in haemodialysis‐dependent CKD (0%), heart failure (8.1%) and non‐dialysis‐dependent CKD (12.8%). Higher prevalence rates were seen in women's health conditions (30.1%), gastrointestinal (40.6%) and neurology subgroups (51.0% and 55.6%, respectively). The authors conclude that appropriate monitoring, particularly after administration of ferric carboxymaltose, is crucial, especially in the rare event of repeated dosing in heart failure patients, to refine management strategies further.^19^
